# Phenotypic and genotypic properties of *Microbacterium yannicii,* a recently described multidrug resistant bacterium isolated from a lung transplanted patient with cystic fibrosis in France

**DOI:** 10.1186/1471-2180-13-97

**Published:** 2013-05-03

**Authors:** Poonam Sharma, Seydina M Diene, Sandrine Thibeaut, Fadi Bittar, Véronique Roux, Carine Gomez, Martine Reynaud-Gaubert, Jean-Marc Rolain

**Affiliations:** 1 Unité de Recherche sur les Maladies Infectieuses et Tropicales Emergents (URMITE), CNRS-IRD, UMR 6236, Méditerranée Infection, Faculté de Médecine et de Pharmacie, Aix-Marseille Université, 27 Bd Jean Moulin, Marseille Cedex 05, 13385, France; 2 Centre de Soins de la mucoviscidose et Centre de transplantation Pulmonaire, Centre Hospitalo-Universitaire Nord, chemin des Bourrelly, Marseille, 13015, France

**Keywords:** Cystic fibrosis, Antibiotic resistance, Multidrug resistant bacteria, *Microbacterium yannicii*

## Abstract

**Background:**

Cystic fibrosis (CF) lung microbiota consists of diverse species which are pathogens or opportunists or have unknown pathogenicity. Here we report the full characterization of a recently described multidrug resistant bacterium, *Microbacterium yannicii*, isolated from a CF patient who previously underwent lung transplantation.

**Results:**

Our strain PS01 (CSUR-P191) is an aerobic, rod shaped, non-motile, yellow pigmented, gram positive, oxidase negative and catalase positive bacterial isolate. Full length 16S rRNA gene sequence showed 98.8% similarity with *Microbacterium yannicii* G72T type strain, which was previously isolated from *Arabidopsis thaliana*. The genome size is 3.95Mb, with an average G+C content of 69.5%. *In silico* DNA-DNA hybridization analysis between our *Microbacterium yannicii* PS01isolate in comparison with *Microbacterium testaceum* StLB037 and *Microbacterium laevaniformans* OR221 genomes revealed very weak relationship with only 28% and 25% genome coverage, respectively. Our strain, as compared to the type strain, was resistant to erythromycin because of the presence of a new *erm* 43 gene encoding a 23S rRNA N-6-methyltransferase in its genome which was not detected in the reference strain. Interestingly, our patient received azithromycin 250 mg daily for bronchiolitis obliterans syndrome for more than one year before the isolation of this bacterium.

**Conclusions:**

Although significance of isolating this bacterium remains uncertain in terms of clinical evolution, this bacterium could be considered as an opportunistic human pathogen as previously reported for other species in this genus, especially in immunocompromised patients.

## Background

Cystic fibrosis (CF) is one of the most common inherited autosomal recessive disease in the Caucasian population. It is due to mutations in the product of the gene encoding the CF transmembrane conductance regulator (CFTR), resulting in chloride channel dysfunction conductance regulator gene [1]. Although CF is a multisystemic disease, the clinical picture is generally dominated by pulmonary involvement, the main cause of morbidity and mortality in this disease. Lung disease is characterized by recurrent and alternative cycles of airway infection and inflammation, leading to bronchiectasis and subsequently to respiratory failure where lung transplantation may constitute the ultimate therapeutic option [2]. Infections in CF patients are considered to be polymicrobial [3]. The pathogens which are traditionally involved include *Pseudomonas aeruginosa, Staphylococcus aureus, Haemophilus influenzae* and *Burkholderia cepacia* complex [4-7]. Many studies have shown that the community of microbes present in the airway of CF patients is more diverse and complex than previously thought [3,8-10]. Many new, emerging and/or multidrug resistant bacteria have been recently reported in CF patients using different technologies including new culture media and molecular methods [3,8,11,12]. In this study, we report the isolation and full description of *Microbacterium yannicii* isolated from the sputum sample from a lung transplanted CF adult patient for which we have recently published the genome sequence [13]. *Microbacterium yannicii* G72T the reference type strain isolated from surface sterilized roots of *Arabidopsis thaliana* was used for comparison [14]. The genus *Microbacterium* was first proposed in 1919 [15]. *Microbacterium* sp. belongs to the family Microbacteriaceae [16,17], order Actinomycetales, class Actinobacteria [17] which comprises mainly aerobic Gram positive bacteria with high G+C content and a peptidoglycan defined by a B-type cross linkage [18]. Based on phylogenetic properties and chemotaxonomic features, the genera *Microbacterium* and *Aureobacterium* were unified to form the redefined genus *Microbacterium* in 1998 [19]. From mid 1990s, the presence of *Microbacterium* was recognized in human clinical specimens [20-22]. However, to the best of our knowledge, bacteria of this genus have never been reported in clinical samples from CF patients. Here, we present a full description of phenotypic and genomic properties of this new bacterium isolated from a CF sputum sample.

### Case report

A 23-year-old woman who has been lung transplanted for CF (heterozygote delta F508/1717-1G genotype) was admitted in emergency in November 2010 in our medical department for acute respiratory failure in the context of uncontrolled CF-related diabetes with ketoacidosis coma. She required rapidly mechanical ventilation support, adapted metabolic adjustment, diabetes management, and nutritional supporting care. Her medical history included long term colonization by multi drug resistant *Pseudomonas aeruginosa* and *Burkholderia multivorans*. She had undergone bilateral lung transplantation when she was 19 years old, and 2 years later, she developed progressive chronic lung allograft dysfunction (CLAD) with a bronchiolitis obliterans syndrome (BOS) stage 3 since the last 6 months and a respiratory insufficiency requiring oxygen supplement 2 months before the admission. The immunosuppressive regimen on admission consisted of tacrolimus (trough level around 8 to 10 ng/ml), mycophenolate mofetil (500 mg twice daily) and azithromycin 250 mg daily for BOS for more than one year. The worsening of respiratory function was associated with the persistence of *Pseudomonas aeruginosa* and *Burkholderia multivorans* colonization along with appearance of *Aspergillus fumigatus*. During hospitalization in the ICU, probabilistic antibiotherapy consisted of an association of ceftazidime, tobramycin and inhaled colistin. After an initial improvement, despite she still required oxygenotherapy device and intermittent noninvasive ventilation support, her respiratory function worsened on January 2011. A sputum sample was collected on January 7th, in which multiresistant *Pseudomonas aeruginosa* and *Burkholderia multivorans* were isolated on chocolate Poly ViteX agar (bioMérieux, Marcy l’Etoile, France) and cepacia agar (AES laboratory, Combourg, France), respectively. An atypical gram positive strain was isolated at 10^5^ CFU/ml on Columbia CNA agar plate. A treatment with ceftazidime, temocillin and inhaled colistin was started again. Her respiratory function continued to deteriorate and she died after 2 months in a septic clinical condition.

## Results

### Phenotypic features

**Figure 1:**
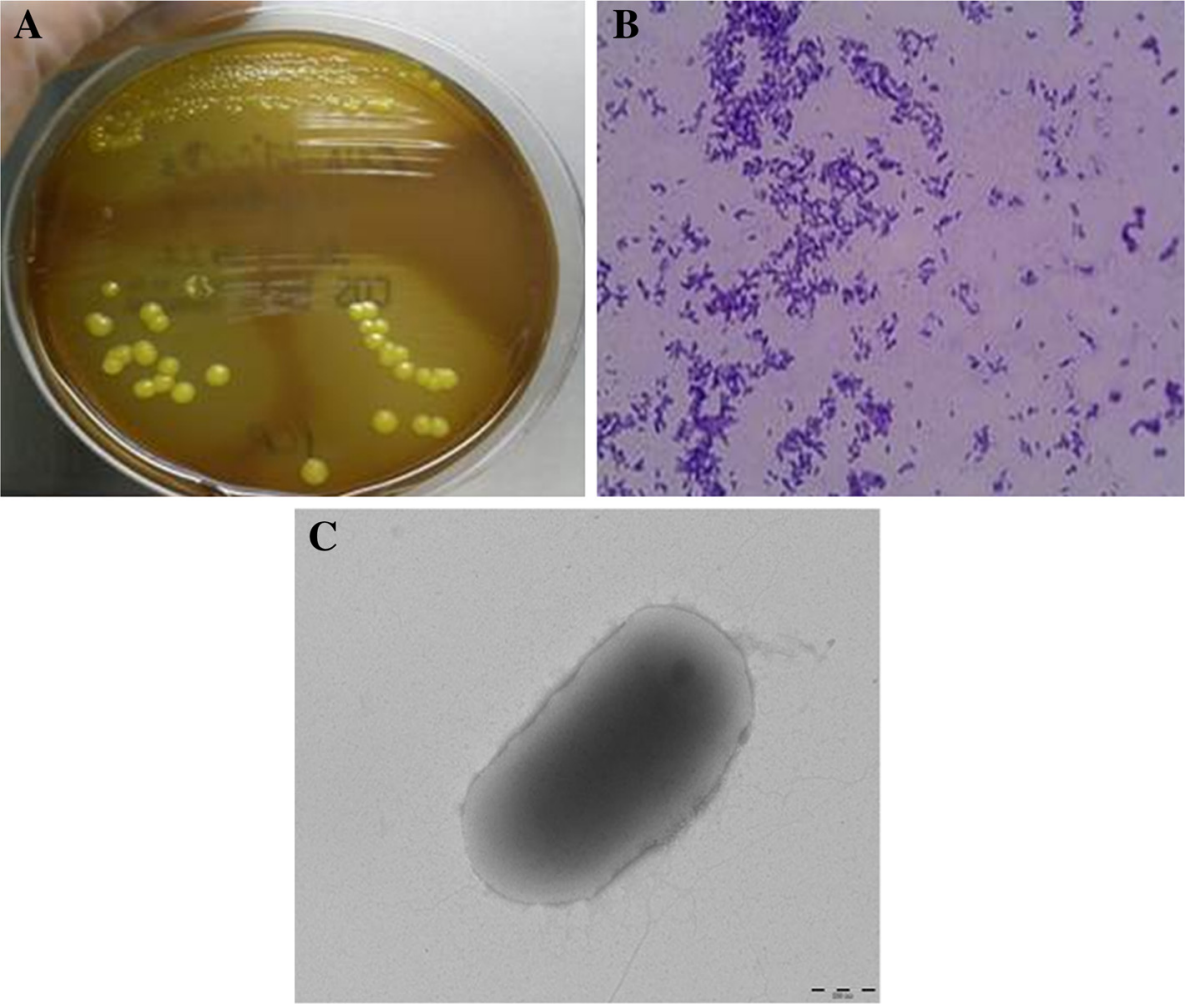
**Colonial morphology, gram staining and transmission electron microscopic image of the CF clinical isolate *Microbacterium yannicii *PS01. A**. CF clinical isolate *Microbacterium yannicii* PS01 was grown on Columbia colistin-nalidixic acid agar with 5% sheep blood (bioMérieux) at 37°C with 5% CO_2._ The colony appeared as yellow, round and smooth. **B**. Gram staining picture of the gram-positive coccobacilli CF clinical isolate “CF *Microbacterium yannicii* PS01*”* viewed at 100X magnification. **C**. Transmission electron microscopy image of *M. yannicii* strain PS01, using a Morgani 268D (Philips) at an operating voltage of 60kV. The scale bar represents 900 nm.

The gram positive strain was isolated on Columbia colistin-nalidixic acid CNA agar with 5% sheep blood (bioMérieux), after 24 hours of incubation at 37°C with 5% CO_2_ (Figure 1A,1B,1C). It also grew on COS medium at 29°C after 24 hours. The colonies are 0.1-0.2 mm in diameter. The isolate was an aerobic, yellow pigmented (Figure 1A), rod-shaped, non-motile, oxidase negative and catalase positive bacterium. This strain was able to grow in microaerophillic atmosphere but not in anaerobic atmosphere. It also grew very weakly at a salt concentration of up to 10% after 48 hours of incubation. As the spectrum for *Microbacterium yannicii* was not available in the Bruker database at the time of our strain isolation, we were not able to identify correctly and after the addition of *Microbacterium yannicii* G72 type strain spectrum in our local database, our strain was identified as *Microbacterium yannicii* with a low score (Score 1.3). Hence, we proceeded with 16SrRNA sequencing for precise identification. Table 1 outlines the results of the commercial phenotypic tests done which includes apiCoryne, apiCH-50 and apiZYM (BioMerieux, Marcy l’Etiole, France) tests to distinguish the CF clinical isolate from five type strains of the genus *Microbacterium* including *Microbacterium yannicii* G72T DSM 23203, *Microbacterium trichothecenolyticum* DSM 8608*, Microbacterium flavescens* DSM 20643 and *Microbacterium hominis* DSM 12509. In apiZYM, the enzymatic reaction for β-glucuronidase was positive for CF *Microbacterium yannicii* PS01 as well as *Microbacterium yannicii* G72T (DSM 23203)*.* Although some of the biochemical tests for our strain yielded results similar to those reported for *M. yannicii* G72 type strain [14], however, we found at least nine differences between our isolate and the type strain that are presented in Table 1 along with comparison to the three other type strains. Antibiotic susceptibility was determined on Columbia agar with 5% sheep blood (COS) (bioMérieux) as per CA-SFM guidelines for *Coryneform* species. Table 2 shows the antibiotic susceptibility pattern of these five strains. The CF clinical strain was resistant to fosfomycin, erythromycin, clindamycin, gentamicin, tobramycin, ciprofloxacin and ofloxacine. The CF clinical isolate was also resistant to trimethoprim-sulfamethoxazole whereas *M. yannicii* G72 type strain was not (Table 2).

**Table 1:** **Comparison of phenotypic characteristics of *M. yannicii *PS01 with closely related species**

**Characteristics**	***CFM.yannicii***	***M.yannicii***	***M.trichothecenolyticum***	***M.flavescens***	***M.hominis***
**Colour of the colony**	Yellow	Yellow	Yellow	Yellow White	Yellow White
**Motility**	No	No	No	No	No
**Growth at 29°C**	Yes	Yes	Yes	Yes	Yes
**Growth at 37°C**	Yes	Yes	Yes	Yes	Yes
**CAT**	**+**	**+**	**+**	**+**	**+**
**OXI**	**-**	**-**	**-**	**-**	**-**
**apiZYM**					
**Esterase lipase**	**+**	**+**	W+	W+	**+**
**Cystine arylamidase**	W+	+	W+	W+	W+
**α-chymotrypsin**	**-**	**-**	**+**	**+**	**-**
**Naphthol-AS-BI-phosphohydrolase**	**-**	**+**	**+**	**-**	**-**
**β-glucuronidase**	**+**	**+**	**-**	**-**	**-**
**α-fucosidase**	**-**	**+**	W+	**-**	**-**
**Assimilation of apiCH50**					
**DARA**	**-**	**+**	**-**	**+**	**-**
**RIB**	**-**	**+**	**-**	**-**	**-**
**DXYL**	**-**	**+**	**+**	**+**	**+**
**GAL**	**-**	**+**	**+**	**-**	**+**
**RHA**	**-**	**-**	**-**	**+**	**+**
**NAG**	**-**	**-**	W+	**-**	**+**
**MEL**	**-**	**+**	**-**	**-**	**-**
**TRE**	**+**	**+**	**-**	**+**	**+**
**INU**	**+**	**-**	**-**	**-**	**-**
**AMD**	**-**	**+**	W+	**-**	**+**
**GLYG**	**-**	**+**	**-**	**-**	**+**
**GEN**	**-**	**+**	**-**	**-**	**+**
**DFUC**	**+**	**+**	**-**	**-**	**-**
**Api CORYNE**					
**Pyr A**	**-**	**-**	**+**	**+**	**-**
**β GUR**	**+**	**+**	**-**	**-**	**-**
**GEL**	**+**	**+**	**-**	**+**	**-**

Phenotypic characteristics Specific phenotypic characteristics of the CF isolate and comparison with closely related *Microbacterium* spp. Strain 1: *M. yannicii* DSM 23203*,* Strain 2: CF *M. yannicii* PS01*,* Strain 3: DSM 8608 *M. trichothecenolyticum,* Strain 4: DSM 20643 *M. flavescens* and Strain 5: DSM 12509 *M.hominis.*

Abbreviation List: + Positive; - Negative; W+ weakly positive; CAT-Catalase; OXI-Oxidase; DARA–D-Arabinose; RIB–Ribose; DXYL–D-Xylose; RHA–L-Rhamnose; NAG–N-AcetylGlucosamine;MEL–D-Mellibiose; TRE–D-Trehalose; INU–Inulin; AMD–Amidon; GLYG–Glycogen; GEN–Gentiobiose; DFUC–D-Fucose; PYRA–Pyroglutamic acid-β-naphthylamide; GUR–Naphthol ASBI-glucuronic acid; GEL–Gelatin (Strictly anaerobic); O–Negative control.

**Table 2:** **Antibiotic susceptibility testing of *M. yannicii *PS01 with closely related species**

**Antibiotic**	**Abr.**	**CF*M.yannicii***	***M.yannicii***	***M.trichothecenolyticum***	***M.flavescens***	***M.hominis***
**Fosfomycin**	FOS50	7/R	7/R	7/R	7/R	7/R
**Chloramphenicol**	C30	S	S	S	16/S	24/S
**Doxycycline**	D30	S	S	S	7/R	7/R
**Erythromycin**	E15	7/R	S	S	7/R	34/S
**Vancomycin**	VA	S	S	S	20/S	14/R
**Clindamycin**	CM5	8/R	S	12/R	7/R	7/R
**Oxacillin**	OX5	20/S	S	7/R	7/R	7/R
**Rifampicin**	RA30	S	S	24/S	28/S	20/S
**Colistin**	CT50	30/S	20/S	20/S	12/R	10/R
**Gentamicin**	GM15	12/R	10/R	14/R	7/R	10/R
**Tobramycin**	TM10	7/R	7/R	7/R	7/R	7/R
**Ciprofloxacine**	CIP5	7/R	15/R	12/R	7/R	20/S
**Ofloxacine**	OFX5	7/R	11/R	10/R	7/R	7/R
**Trimethoprim-Sulfamethoxazole**	SXT	7/R	31/S	24/S	S	S
**Amoxicillin**	AX25	S	S	S	S	20/S
**Imipenem**	IMP10	S	S	S	S	S
**Ceftazidime**	CAZ30	S	7/R	7/R	7/R	16/S
**Ticarcilline**	TIC75	S	S	7/R	7/R	12/R
**Cefoxitin**	FOX30	S	20/S	7/R	16/S	26/S
**Ceftriaxone**	CRO30	S	S	24/S	7/R	S
**Amoxicillin-Clavulinic acid**	AMC30	S	S	S	S	S

Antibiotic susceptibility testing of CF clinical *M. yannicii* PS01 isolate and *M. yannicii* DSM 23203, *M. flavescens, M. trichothecenolyticum* and *M. hominis* reference strains.

*S* sensitive, *R* resistant, Numbers given in mm.

### Genotypic features

**Figure 2:**
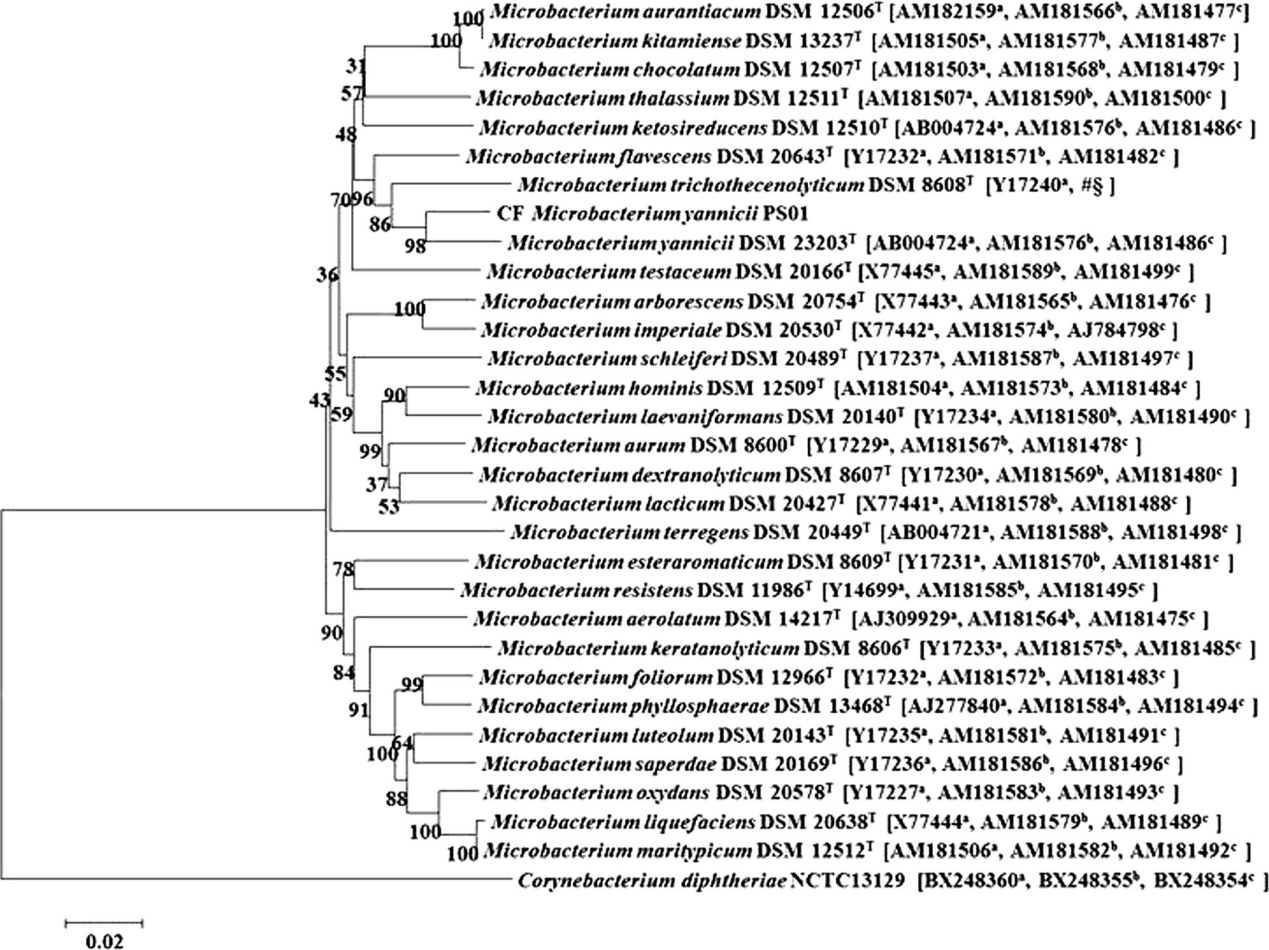
**Concatenated phylogenetic tree of *Microbacterium *species using NJ method.** Concatenated phylogenetic tree based on 16SrRNA-*rpoB-gyrB* sequence highlighting the phylogenetic position of CF *Microbacterium yannicii* PS01*. Corynebacterium diphtheriae* was used as an out group. Sequences were aligned using CLUSTALX and Phylogenetic inferences obtained using Neighbor joining method within Mega 5 software. Bootstrap values are expressed by percentage of 1000 replicates with Kimura 2 parameter test and shown at the branching points. The branches of the tree are indicated by the genus and species name of the type strains followed by the NCBI Gene accession numbers: a: 16SrRNA; b: *rpo*B; c: *gyr*B. (# *rpo*B and § *gyr*B sequence of *M. trichothecenolyticum* was obtained for this study).

The 16S rRNA sequence of our isolate Strain PS01 showed 98.8% similarity with *Microbacterium yannicii* G72T strain (DSM23203) (GenBank accession number FN547412), 98.7% with *Microbacterium trichothecenolyticum*, and 98.3% similarity with both *Microbacterium flavescens* and *Microbacterium hominis*. Based on 16S rRNA full length gene sequence (1510 bp), our isolate was identified as *Microbacterium yannicii.* Partial *rpoB* sequences (980 bp) as well as partial gyrB sequences were also determined for the four strains and a concatenated phylogenetic tree was constructed to show the phylogenetic position of CF *Microbacterium yannicii* PS01 (Figure 2).

### Genome analysis and comparison

The genome of CF *Microbacterium yannicii* (Strain PS01, CSUR Reference No.P191) was sequenced and the draft genome sequence has been deposited in EMBL under the accession number CAJF01000001-CAJF01000067 [13]. The genome exhibits a total size of 3,952,501 bp and a G+C content of 69.5% (Table 3). We performed *in silico* DNA - DNA hybridization of the whole genome of CF *Microbacterium yannicii* against the two available genomes in this genus i.e. *Microbacterium testaceum* StLB037 and *Microbacterium laevaniformans* OR221 and the overall results are presented in Table 3. At E-value 1.00e-5, the species coverage in *Microbacterium testaceum* StLB037 and *Microbacterium laevaniformans* OR221 was only 28% and 25.05%, respectively (Table 3). The numbers of proteins with no similarity in comparison to CF *Microbacterium yannicii* were 882 and 988 and with similarity up to 80% were 598 and 580 of *Microbacterium testaceum* StLB037 and *Microbacterium laevaniformans* OR221, respectively (Table 3). We analyzed the resistome of CF *M. yannicii* PS01 and found that there were 11 ORFs corresponding to Beta-lactamase family proteins, 5 ORFs corresponding to Aminoglycoside phosphotransferase family proteins that could explains the resistance of this isolate to aminoglycosides, 1 ORF corresponding to a macrolide efflux protein family and a new *erm* gene encoding a 23S rRNA N-6-methyltransferase that could explain the resistance to erythromycin (Table 2 and Table 4). *Microbacterium yannicii* G72T reference strain was susceptible to erythromycin and after designing primers targeting the new *erm* gene we found that this reference strain do not contain this gene as PCR was only positive for our CF isolate (data not shown). We also found mutations in *gyrA* (Ser83Ala) and *parC* (Ser80Ala) that were likely the cause of resistance against fluoroquinolone compounds (Table 2 and Table 4). Resistance to trimethoprim-sulfamethoxazole was likely due to the presence of a DHPS encoding gene (Table 4). We also found 17 ORFs for multidrug efflux transporters such as ion channels, multidrug ABC transporters, amino acid transporters, and major facilitator superfamily proteins which could explain the resistance to other antibiotics (Table 4).

**Table 3:** **General features of *M.yannicii *PS01 genome in comparison with *M. testaceum *StLB037 and *M. laevaniformans *OR221 genomes**

**Species**	**Database accession number**	**Genome size (bp)**	**%GC content**	**No. of CDS**	**No. of RNA**	**Alignment length (bp)**	**Y**^**∆**^**= 0%**	**Y**^**∆**^**> 80%**
						**(cut-off E-value 1.00e-5)**^**β**^	**Id**	**Id**
***M. yannicii*PS01**	CAJF01000001- CAJF01000067 ^*α*^	3,952,501	69.54	3772	56	-	-	-
***M. testaceum*StLB037**	AP012052	3,982,034	70.28	3795	58	1,106,788 (28%)	882	598
***M. laevaniformans*OR221**	AJGR01000001- AJGR01000535 ^α^	3,427,400	68.03	3294	72	989,933 (25.05%)	988	580

Table of comparison of *M. yannicii* PS01genome details in comparison with *M. testaceum* StLB037 and *M. laevaniformans* OR221 genomes.

^α^, genome sequences were deposited in Whole Genome Sequence (WGS), 67 contigs for *M. yannicii* and 535 contigs for *M. laevaniformans*;

^β^, the “*In silico*” DNA-DNA hybridization of *M. yannicii* PS01 genome against *M. testaceum* StLB037 and *M. laevaniformans* OR221 genomes, in parenthesis, the percentage of coverage with respect to *M. yannicii* genome; ^∆^, Number of *M. yannicii* proteins with any similarity and with similarity up to 80%.

**Table 4:** **Antibiotic resistance genes in *M.yannicii *PS01 genome**

**Antibiotic class**	**Gene name**	**Size (aa)**	**Functions**	**Best blast hit organism in Genbank**	**% aa identity**	**E-value**
**Beta-lactams**	***ampC***	323	Beta-lactamase class C	*Isoptericola variabilis* 225	56.5	5.00E-114
	***ampC***	422	Beta-lactamase class C	*Microbacterium testaceum* StLB037	54	1.00E-123
	***ampC***	364	Beta-lactamase class C	*Paenibacillus mucilaginosus* KNP414	37.8	3.00E-59
	***ampC***	338	Beta-lactamase class C	*Arthrobacter aurescens* TC1	41.9	4.00E-67
	***-***	558	Predicted hydrolase of the metallo-beta-lactamase superfamily	*Microbacterium laevaniformans* OR221	88.8	0
	***-***	212	Predicted Zn-dependent hydrolases of the beta-lactamase fold	*Microbacterium testaceum* StLB037	66.9	2.00E-100
	***elaC***	290	Metal-dependent hydrolases of the beta-lactamase superfamily III	*Saccharomonospora paurometabolica* YIM 90007	46.6	2.00E-74
	***penP***	279	Beta-lactamase class A	*Microbacterium testaceum* StLB037	77.3	7.00E-146
	***-***	615	Beta-lactamase domain protein	*Kribbella flavida* DSM 17836	44.2	3.00E-148
	***-***	626	Beta-lactamase domain protein	*Mycobacterium rhodesiae* JS60	66.8	0
	***-***	524	Zn-dependent hydrolase of the beta-lactamase fold	*Microbacterium testaceum* StLB037	83.8	0.00E+00
**Aminoglycoside**	***aph***	51	Aminoglycoside phosphotransferase	*Microbacterium laevaniformans* OR221	72	2.00E-17
	**-**	435	Predicted aminoglycoside phosphotransferase	*Microbacterium testaceum* StLB037	61	3.00E-130
	**-**	292	Aminoglycoside phosphotransferase	*Micromonospora lupini* str. *Lupac* 08	55.9	3.00E-95
	**-**	308	Aminoglycoside phosphotransferase	*Streptosporangium roseum* DSM 43021	43.8	7.00E-71
	**-**	350	Aminoglycoside phosphotransferase	*Cellulomonas fimi* ATCC 484	60.4	1.00E-125
**Macrolides**	**-**	461	Macrolide-efflux protein	*Beutenbergia cavernae* DSM 12333	65.4	2.00E-166
**Fluoroquinolones**	***gyrA*mutated: S83A**	883	DNA gyrase subunit A (EC 5.99.1.3)	*Microbacterium testaceum* StLB037	87.7	0
	***parC*mutated: S80A**	819	Topoisomerase IV subunit A (EC 5.99.1.-)	*Microbacterium testaceum* StLB037	82.7	0
**Sulfamides**	***dhps***	281	Dihydropteroate synthase	*Microbacterium laevaniformans* OR221	71.6	2.00E-122
**Multidrug Efflux pumps**	***corC***	450	Magnesium and cobalt efflux protein	*Microbacterium testaceum* StLB037	78.4	0
	***kefA***	373	Potassium efflux system	*Microbacterium laevaniformans* OR221	74.7	0
	**-**	548	Putative MFS Superfamily multidrug efflux transporter	*Nocardia cyriacigeorgica* GUH-2	72.8	0
	**-**	513	putative efflux MFS permease	*Microbacterium laevaniformans* OR221	78.8	0
	**-**	212	Putative threonine efflux protein	*Microbacterium testaceum* StLB037	61.6	1.00E-80
	**-**	1275	RND multidrug efflux transporter; Acriflavin resistance protein	*Microbacterium laevaniformans* OR221	78.1	0
	**-**	426	antibiotic efflux protein	*Microbacterium laevaniformans* OR221	83.5	0
	**-**	526	probable multidrug resistance transporter, MFS family	*Cellulomonas fimi* ATCC 484	68.8	0
	**-**	474	Inner membrane component of tripartite multidrug resistance system	*Arthrobacter aurescens* TC1	68.2	0
	**-**	354	ABC-type multidrug transport system, ATPase component	*Saccharopolyspora erythraea* NRRL 2338	58.8	1.00E-119
	***bcr/cflA***	417	Multidrug resistance transporter, Bcr/CflA family	*Brachybacterium paraconglomeratum* LC44	68.5	1.00E-154
	**-**	519	multidrug resistance protein	*Arthrobacter aurescens* TC1	54.2	8.00E-177
	**-**	332	ABC-type multidrug transport system, ATPase component	*Microbacterium laevaniformans* OR221	72.2	6.00E-142
	**-**	264	ABC-type multidrug transport system, ATPase component	*Microbacterium testaceum* StLB037	75	1.00E-143
	**-**	303	ABC-type multidrug transport system, ATPase component	*Paenibacillus curdlanolyticus* YK9	59.5	7.00E-110
	**-**	273	ABC-type multidrug transport system, permease component	*Paenibacillus curdlanolyticus* YK9	67.7	3.00E-121
	**-**	306	ABC-type multidrug transport system, ATPase component	*Clavibacter michiganensis* subsp. *michiganensis* NCPPB 382	60.8	3.00E-107

General features of CF *M. yannicii* PS01 resistome showing the antibiotic resistance genes present and percentage of identity with best blast hit organism.

## Discussion

**Figure 3:**
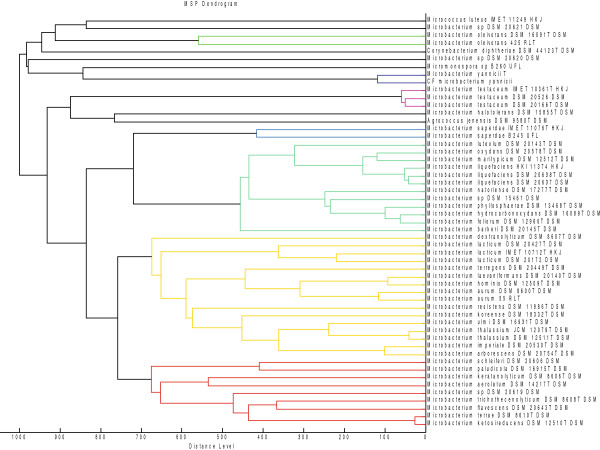
**MSP Dendrogram based on different *Microbacterium *species hierarchy along with other species of the genus *Microbacterium *(reference spectra obtained from Bruker database) upon the addition of *Microbacterium yannicii *G72 type strain and CF *Microbacterium yannicii *PS01.** (* Spectra generated).

Genus *Microbacterium* belongs to the Microbacteriaceae family, which contains species highly related by 16S rRNA gene sequence that are difficult to identify at the species level [19]. In this genus, the only available genomes before our previous work [23] were those of *Microbacterium testaceum* StLB037 and [23] and *Microbacterium laevaniformans* OR221 [24]. We used a polyphasic taxonomic approach for the precise identification of our new species. Firstly, MALDI-TOF-MS was used for the identification of the bacterium. MALDI-TOF-MS, a rapid and reliable method to identify bacterial isolates at the species and subspecies level [25,26] was used for the identification of this bacterium. Although initially, our strain was only identified at the genus level, it was correctly identified as *Microbacterium yannicii* at the species level when spectrum from the reference strain was added to the database (Figure 3). We performed apiZYM, apiCH50, apiCoryne and antibiotic susceptibility phenotypic tests to compare our strain to *Microbacterium yannicii* G72 type strain as well as to other closely related species (*Microbacterium trichothecenolyticum*, *Microbacterium flavescens* and *Microbacterium hominis*). In these tests, we have found only few differences between our strain and the type strain. For example we found that the reference strain was susceptible to erythromycin whereas our strain was not, and this was likely due to the presence of a 23S rRNA methyltransferase in the genome of our strain that was absent in the reference strain. These differences may be explained by the different lifestyle of the two strains, one living in roots and the other in human respiratory tract leading to different genomic repertoires that can produce alternative or atypical phenotypes that are better adapted to their environment as recently exemplified in *Enterobacter aerogenes*[27]*.* Finally, whole genome sequence analysis of our strain allows us to fully characterize this new species including the genetic determinants associated with its specific antibiotic resistance phenotype likely acquired from different sources. *In silico* DNA-DNA hybridization of the genome of CF *Microbacterium yannicii* against the two other available genomes (*Microbacterium testaceum* StLB037 and *Microbacterium laevaniformans* OR221) was very low (≤ 70%). This was similar to DNA-DNA hybridization experiments reported in the seminal paper on the description of *Microbacterium yannicii* G72T species by Karojet *et al.* who showed a genetic relatedness of only 15.9%, 31.2%, and 45.1% between reference strain *Microbacterium yannicii* G72 and *Microbacterium hominis, Microbacterium insulae,* and *Microbacterium trichothecenolyticum*, respectively [14]. As all the organ transplant recipients, our patient was immunocompromised, with an over immunosuppressive regimen containing a long macrolide therapy in the context of chronic lung allograft dysfunction, such conditions with might play a crucial role in the development of *Microbacterium* spp. infection or colonization. Indeed *Microbacterium* spp. have been described as a causative agent of infections in immunocompromised patients such as, cancer patients [28,29], endophthalmitis patients [21], interstitial pulmonary infection after heart transplantation [30], bone marrow transplant recipients [31], and bacteremia [32-34]. To the best of our knowledge, such infection with *Microbacterium* spp has not been previously described in the double context of lung transplantation and in cystic fibrosis. *Microbacterium* spp. have been isolated from clinical specimens including blood culture, superficial wounds, pleural fluid, sinus aspirate, bone infection, endophthalmitis, dialysis fluid, lymph node, catheter tip, knee puncture fluid, wound swab, urine, gall bladder, throat swab, prosthetic hip infection, conjuctival swab, tracheal secretion and urethral swab [35]. The source of this bacterium in our patient was also undetermined but in our opinion, plants or vegetables may be a potential source of transmission in CF patients as well as a possible person to person transmission from another patient. Bacteria of the genus *Burkholderia, Pandoraea,* or *Pseudomonas* for example, which are known to be frequently recovered in the respiratory tract of CF patients, are also endophytic bacteria in plants. There results reinforce the hypothesis that plant associated environments may act as niche for putative opportunistic human pathogenic bacteria [36].

## Conclusions

Our results showed that CF *Microbacterium yannicii*, which has previously been isolated from *Arabidopsis thaliana* roots, has never been reported from a human clinical specimen and its pathogenicity in this context is unknown. Studies have shown that bacteria from this genus have been associated previously with infections, predominantly in immunocompromised patients; however, the isolation of *Microbacterium yannicii* is unclear if it could have been the result of a specific exacerbation observed in this patient. In our study, the patient received immunosuppressive therapy since her lung transplantation. Because the patient was also chronically colonized by other well-known pathogens, it is difficult to establish the true significance of isolating this bacterium in terms of clinical evolution. Hence, it is hypothesized that this bacterium could be considered as an opportunistic human pathogen in immunocompromised patients but this should be further investigated in the future.

## Methods

### Bacterial isolate and identification

*Microbacterium yannicii* G72T reference strain (DSM23203) [14] was used as a control for the comparison of phenotypic and genotypic properties of our strain. Our CF strain was isolated on Columbia CNA agar plate (bioMérieux), and was identified by Matrix assisted Laser desorption and ionization time-of-flight mass spectrometry (MALDI TOF-MS) using a Microflex machine (Bruker Daltonics)*.* The biochemical tests were performed on the commercially available apiCoryne, apiCH-50 and apiZYM test strips (BioMerieux, Marcy l’Etiole, France) according to manufacturer’s i0n1str0uctions.

### Antibiotic susceptibility test

Antibiotic susceptibility was determined on Columbia agar with 5% sheep blood (COS) (bioMérieux) by disk diffusion method as per CA-SFM guidelines for *coryneform* species and the susceptibility results were interpreted according to the recommendations of the “Comité de l’Antibiogramme de la Société Française de Microbiologie (CA-SFM)” (http://www.sfm-microbiologie.org/).

### PCR and sequencing

To investigate the phylogenetic position of this strain, 16S rRNA, *rpo*B and *gyr*B genes were amplified and sequenced with Big Dye Terminator reagents (Applied Biosystems) ABI 3730 Automated Sequencer and the sequences were blasted against the GenBank database. The sequence of the primers used in this study are 16SrRNA F-5′-AGAGTTTGATCCTGGCTCAG-3′, 16SrRNA R-5′-ACGGCTACCTTGTTACGACTT-3′, MY *rpoB* F-5′-AAGGGMACSTTCGTCATCAA-3′, MY *rpoB* R-5′-CGATCAGACCGATGTTCGGG-3′, MY*gyrB* F-5′-GASSGCSTTCCTSAACAAGG-3′and MY*gyrB* R-5′-GCNCGGAASCCCTCYTCGTG-3′. Sequence alignment was performed using CLUSTAL X, and concatenated phylogenetic tree was constructed using MEGA 5 software (Molecular Evolutionary Genetic Analysis, vers.5, 2011) using neighbor joining tree method and 1000 bootstrap replications [37].

### Genome

The genome of this strain was sequenced using Genome Sequencer Titanium (454 Life Sciences, Branford, CT) and reported recently [13]. All contigs from genome assembly process were submitted to online bioserver “RAST server: Rapid Annotation using Subsystems Technology (http://www.theseed.org)” [38] to predict protein-encoding genes, rRNA and tRNA sequences, and assigned functions to these genes. Predicted proteins were compared against Non Redundant (nr) GenBank database using BLASTP (e-value 10^E-8^; identity ≥30%; coverage ≥50%) and COG databases of the National Center for Biotechnology Information (NCBI) (http://www.ncbi.nlm.nih.gov). tRNA and rRNA genes were also verified on tRNAscan-SE Search Server (http://lowelab.ucsc.edu/tRNAscan-SE) and RFAM (http://rfam.sanger.ac.uk) respectively. Genome comparison was performed by “*in silico”* DNA-DNA hybridization using BlastN analysis in a local bioserver to determine the full-length alignment between two genome sequences and the coverage percentage using the cut-off stringency of E-value at 1.00e-5 [30].

## Competing interests

The authors declare that they have no competing interests.

## Authors’ contributions

PS carried out all the experiments and wrote the manuscript. SMD carried out the genomics study. ST and CG contributed the case report. VR helped in analyzing data. FB and MRG critically revised the manuscript. JMR conceived the idea, analyzed the data and helped to draft the manuscript. All authors read and approved the final manuscript.
